# Classification and Quantification of Physical Therapy Interventions across Multiple Neurological Disorders: An Italian Multicenter Network

**DOI:** 10.3390/jcm12206483

**Published:** 2023-10-12

**Authors:** Thomas Bowman, Fabiola Giovanna Mestanza Mattos, Silvia Salvalaggio, Francesca Marazzini, Cristina Allera Longo, Serena Bocini, Michele Gennuso, Francesco Giuseppe Materazzi, Elisa Pelosin, Martina Putzolu, Rita Russo, Andrea Turolla, Susanna Mezzarobba, Davide Cattaneo

**Affiliations:** 1 IRCCS Fondazione Don Carlo Gnocchi, 20148 Milan, Italy; davide.cattaneo@unimi.it; 2 Department of Pathophysiology and Transplantation, Università degli Studi di Milano, 20100 Milan, Italy; fabiola.mestanza@unimi.it; 3 Laboratory of Computational Neuroimaging, IRCCS San Camillo Hospital, Via Alberoni 70, 30126 Venice, Italy; silvia.salvalaggio@hsancamillo.it; 4 Padova Neuroscience Center, Università degli Studi di Padova, via Orus 2/B, 35131 Padova, Italy; 5 AIAS ETS Milano, Piazza Comunale 21, 20048 Pantigliate, Italy; marazzini@aiasmilano.it; 6 Department of Rehabilitation, San Carlo Borromeo Hospital, 20153 Milan, Italy; cristina.alleralongo@asst-santipaolocarlo.it (C.A.L.); rita.russo@asst-santipaolocarlo.it (R.R.); 7 Division of Physical and Rehabilitation Medicine, Fondazione Opera San Camillo, Presidio di Torino, 10131 Torino, Italy; serena.bocini@camilliani.net; 8 Department of Neurological Sciences, Neurorehabilitation Clinic, AOU Delle Marche, 60126 Ancona, Italy; michele.gennuso@ospedaliriuniti.marche.it; 9 Montecatone Rehabilitation Institute, 40026 Imola, Italy; francesco.materazzi@montecatone.com; 10 Department of Biotechnological and Applied Clinical Sciences (DISCAB), University of L’Aquila, 67100 L’Aquila, Italy; 11 Department of Neuroscience, Rehabilitation, Ophthalmology, Genetics, Maternal and Child Health, University of Genoa, 16132 Genoa, Italy; elisa.pelosin@unige.it (E.P.); susanna.mezzarobba@unige.it (S.M.); 12 IRCCS Ospedale Policlinico San Martino, IRCCS, 16132 Genoa, Italy; 13 Department of Experimental Medicine (DIMES), Section of Human Physiology, University of Genoa, Viale Benedetto XV/3, 16132 Genoa, Italy; martina.putzolu@unige.it; 14 Department of Biomedical and Neuromotor Sciences (DIBINEM), Alma Mater Studiorum—Università di Bologna, 40138 Bologna, Italy; andrea.turolla@unibo.it; 15 Unit of Occupational Medicine, IRCCS Azienda Ospedaliero-Universitaria di Bologna, 40138 Bologna, Italy; 16 Azienda Sanitaria Universitaria Giuliano Isontina (ASUGI), 34128 Trieste, Italy

**Keywords:** physical therapy, neurorehabilitation, classification, quantification, prevalence, effectiveness, Parkinson’s disease, multiple sclerosis, stroke

## Abstract

Despite their relevance in neurorehabilitation, physical therapy (PT) goals and interventions are poorly described, compromising a proper understanding of PT effectiveness in everyday clinical practice. Thus, this paper aims to describe the prevalence of PT goals and interventions in people with neurological disorders, along with the participants’ clinical features, setting characteristics of the clinical units involved, and PT impact on outcome measures. A multicenter longitudinal observational study involving hospitals and rehabilitation centers across Italy has been conducted. We recruited people with stroke (*n* = 119), multiple sclerosis (*n* = 48), and Parkinson’s disease (*n* = 35) who underwent the PT sessions foreseen by the National Healthcare System. Clinical outcomes were administered before and after the intervention, and for each participant the physical therapists completed a semi-structured interview to report the goals and interventions of the PT sessions. Results showed that the most relevant PT goals were related to the ICF activities with “walking” showing the highest prevalence. The most used interventions aimed at improving walking performance, followed by those aimed at improving organ/body system functioning, while interventions targeting the cognitive–affective and educational aspects have been poorly considered. Considering PT effectiveness, 83 participants experienced a clinically significant improvement in the outcome measures assessing gait and balance functions.

## 1. Introduction

The prevalence of neurological disorders, resulting from disease or injury of the nervous system, is increasing worldwide. It is estimated that up to 1 billion people worldwide are affected by neurological disorders, constituting 6.3% of the global burden of disease [1]. The activities of daily life (ADL) of people with neurological disorders (PwND) can be affected by several symptoms, including physical, cognitive, behavioral, and communication deficits [2]. This significant disability burden requires a specialized approach, called neurorehabilitation. Neurorehabilitation is a multidisciplinary care program coordinating the efforts of professionals such as neurologists, nurses, physical therapists, and allied health professionals with the aim of stimulating neuroplasticity and the recovery of functional abilities [3]. In this context, physical therapy (PT) interventions help PwND increase strength, mobility, balance, walking, and coordination so they can improve participation in ADL [4]. As defined by the American Physical Therapy Association (APTA), PT consists of the application of therapeutic interventions to promote, maintain, or restore the physical and physiological well-being of an individual [5]. A whole set of therapeutic interventions defines the PT process, and its comprehension is relevant to determining the relationship between interventions and outcomes. According to the World Physiotherapy Association [6], there are more than 1,917,615 active physical therapists in the world, of which 67,656 are in Italy, and these numbers are growing, given the increasing demand for PT due to the gradual aging of the population and the increasing prevalence of neurological disorders. Despite its importance, an overall picture of PT for PwND is missing, and the whole set of PT interventions and their relationship with the outcomes is not well defined. Several International Clinical Guidelines about PT have been published to provide the best practice and to improve treatment standards for PwND [7]. However, even if many health professionals are aware of clinical guidelines, there are multiple barriers compromising their implementation in clinical practice. In particular, poor organizational support, low level of awareness, familiarity, or confidence in a particular evidence-based therapy, the lack of univocal and non-redundant terminology, the diffusion of myths and misconceptions, as well as a clinical practice that is not always evidence-based, have hindered a reliable determination of the contributions of each PT intervention to the rehabilitation outcomes [8,9]. Actually, several authors [10,11] have asserted that failure to identify PT intervention is a serious omission that prevents a correct understanding of PT effectiveness. Due to this, PT has been defined as a “black box”, and only a few researchers [12,13,14,15] investigated PT components. According to this, there is a need for studies investigating PT application in everyday clinical practice to understand its effectiveness, the contribution of different types of interventions, and their components. PT efficacy has been widely reported and assessed by randomized control trials (RCT) and systematic reviews [16,17,18,19,20]. However, RCT usually investigated efficacy under ideal and controlled circumstances (e.g., strict inclusion and exclusion criteria), while rehabilitation performances under “real-world” conditions, known as intervention effectiveness, have been poorly investigated by pragmatic trials [21]. Research on rehabilitation effectiveness is necessary to examine relationships between interventions and outcomes and to overcome the limited generalizability of RCT [22]. Practice-based evidence (PBE) research is the most appropriate methodology to investigate the heterogeneity of patients, interventions, and outcomes seen in real-world clinical settings [23,24,25,26,27]. PBE study designs comprise different elements to improve the value of clinical research: inclusion of different populations, comparison of clinically relevant alternative therapies, sample recruitment from heterogeneous settings, and data collection including a broad range of health outcomes. The application of PBE methodology in neurorehabilitation involves the participation of clinicians engaged in the care process requiring the identification of rehabilitation components and detailed documentation of each intervention provided [23]. Therefore, in line with the previous considerations, this paper aims to provide an overall picture of PT for PwND in Italy according to the PBE methodology. We collected data from a multicenter network involving hospitals and rehabilitation centers all over Italy, to study and describe the clinical setting and characteristics of the centers involved, the prevalence of PT goals and interventions, and their impact on outcome measures.

## 2. Materials and Methods

### 2.1. Study Population, Setting, and Design

All the participants in this longitudinal prospective cohort study were enrolled in a multicenter network from January 2018 to January 2022. The multicenter network involved hospitals and rehabilitation centers all over Italy:

- Associazione Italiana Assistenza Spastici Onlus—Milan
- AOU Ospedali Riuniti Torrette—Ancona
- Azienda Sanitaria Universitaria Giuliano Isontina—Trieste
- IRCCS Fondazione Don Carlo Gnocchi, Centro di Santa Maria Nascente—Milan
- IRCCS Ospedale San Camillo—Venice
- IRCCS Ospedale Policlinico San Martino—Genoa
- Istituto Don Orione—Pescara
- Presidio Sanitario San Camillo—Turin
- ASST Santi Paolo e Carlo—Milan

The study was registered on clinical trial.gov (ID: NCT04386863) and was approved by the Regional Ethical committees. The study was conducted in agreement with the Declaration of Helsinki, and all participants provided written informed consent prior to the longitudinal trial.

### 2.2. Eligibility Criteria

A convenience sample was recruited for the study in the centers involved. We included subjects with a diagnosis of multiple sclerosis [28], Parkinson’s disease (MDS Criteria) [29], and stroke (ischemic or haemorrhagic stroke confirmed according to World Health Organization criteria) [30]. All subjects were from the rehabilitation services of the National Health System (NHS) and performing at least 10 physical therapy sessions was included. All subjects with less than 18 years or those not able to understand the study protocol were excluded.

### 2.3. Experimental Procedures

**Figure 1:**
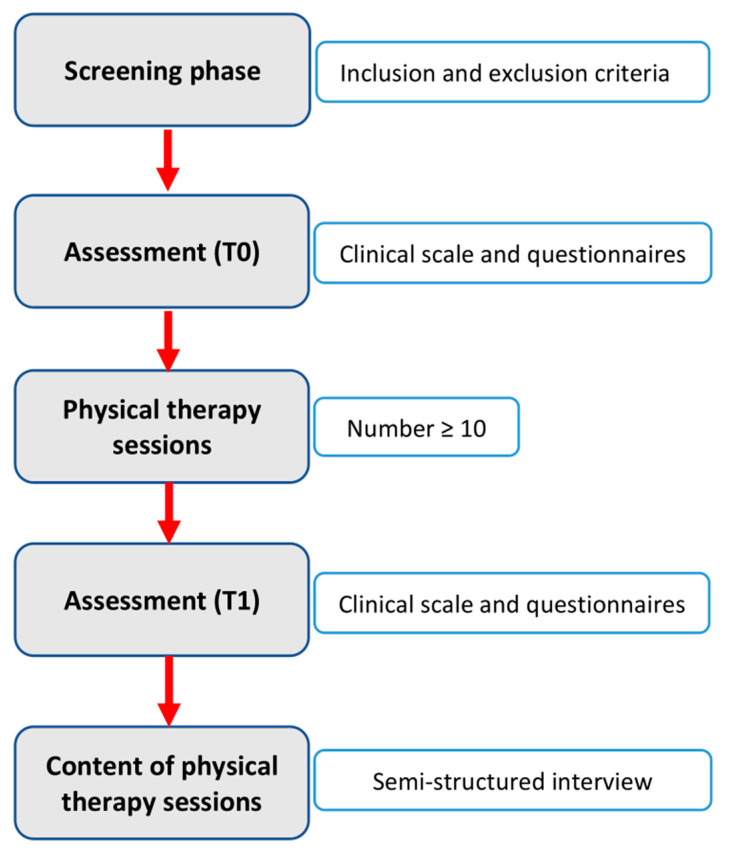
Experimental Procedures (Flow Chart).

All participants underwent the PT sessions foreseen by the NHS and were clinically assessed before (T0) and after (T1) (Figure 1). No follow-up evaluation was performed. All the clinical evaluations were performed by an experienced clinical researcher not involved in the PT sessions.

The physical therapist assigned to each participant completed a semi-structured interview called “PT Interventions Form” (Figure S1). To reduce the burden of assessments, we asked physical therapists to fill out the “PT Intervention Form” only at the end of the rehabilitation program. The semi-structured interview was used to collect information about PT goals and interventions across different neurological populations. 

### 2.4. Assessment (Outcome Measures)

#### 2.4.1. Lower-Limb Function:

Change in two-minute walking test (2MWT): this walking test is used to evaluate functional exercise capacity. Subjects are instructed to walk as fast as possible in 2 min, along a 30 m walkway, and the total distance walked is recorded [31].

Change in modified Dynamic Gait Index (mDGI): The modified Dynamic Gait Index is a clinical outcome measure to assess the ability to modify and adapt gait and balance during complex walking tasks. Higher scores indicate better performances [32].

#### 2.4.2. Upper-Limb Function:

Change in Box and Blocks test (BBT): The BBT measures unilateral gross manual dexterity. The test consists of a box with a partition in the middle and is scored by counting the number of blocks carried over the partition from one compartment to the other during the one-minute trial [33].

#### 2.4.3. Disability and ADL:

Change in Modified Barthel Index (MBI): The MBI is a measure of physical disability used widely to assess behavior relating to ADL for subjects with disabling conditions. Higher scores indicate a low level of disability [34].

#### 2.4.4. PT Intervention Contents

The “PT Interventions Form” is a semi-structured interview developed to be filled out by the physical therapist at the end of PT sessions (Figure S1). The “General Information” section collected demographic characteristics including date of completion, treatments’ parameters, dose (session frequency and duration), rehabilitation setting (inpatient/outpatient), and concomitant therapies, e.g., speech therapy or occupational therapy. The “PT goals” section contained a list of 18 goals divided into impairments, activities, and participation domains according to the International Classification of Functioning, Disability, and Health categories from generic, rehabilitation, and neurological core sets as reported in Table S1 [35]. Physical therapists could specify up to three therapeutic goals with a level of priority. The “Interventions” section comprised a list of 22 interventions to specify the PT contents. In this section, physical therapists indicated up to five types of interventions provided to achieve each therapeutic goal. The amount of time spent per intervention in percentage was also requested along with the total intervention time. The list of goals and interventions included in the PT intervention form have been selected through an iterative process including the analysis of the literature and the experts’ opinion. The final list contained the most relevant goals and interventions according to the experts’ teamwork. Moreover, in this section, physical therapists could manually enter interventions if not on the list, to collect any missing interventions. Finally, a legend describing the key elements of the PT Interventions form was included to guide physical therapists in completing the form.

### 2.5. Statistical Analysis

Descriptive analyses were carried out. The data in the tables are presented as mean (standard deviation) and as a percentage. The prevalence of goals and interventions was calculated by considering the number of hours, and then the percentages in relation to the total treatment time were calculated. Furthermore, to establish the intervention effectiveness, subjects were classified as improved if they reached the minimally clinically important difference (MCID) value in the clinical outcome measures. The MCID corresponds to the minimum achievable improvement on a clinical scale that is perceived as relevant by the patient himself [36]. If the MCID value was not defined in the literature for an outcome measure, it was decided to classify as improved subjects who had at least 20% improvement at T1 on the clinical outcome measures compared to T0 [37,38].

## 3. Results

### 3.1. Characteristics of the Clinical Unit

A total of nine clinical units were included in the study, six hospitals, and three rehabilitation centers. Table 1 shows the number of subjects recruited for each clinical unit, including pathology and the number of inpatient or outpatient subjects.

### 3.2. Demographic and Clinical Characteristics of the Sample

In total, 202 participants (mean age: 65.9 ± 13 years; 96 Female; 106 Male), with different neurological disorders (119 people with stroke (PwST), 48 people with multiple sclerosis (PwMS), and 35 people with Parkinson’s disease (PwPD) were recruited, and the demographic characteristics of the sample are shown in Table 2.

Clinical characteristics at baseline of the whole sample and subgroups are shown in Table 3.

### 3.3. Prevalence of PT Goals 

**Figure 2:**
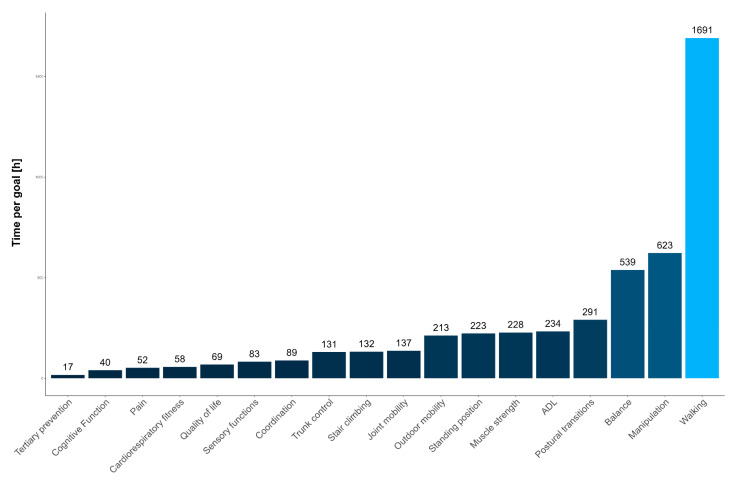
Number of hours per PT goals in PwND.

A total of 4850 h of PT were analyzed in PwND. The average duration of each PT program was approximately (23 ± 17) hours of intervention with an average duration of the single session of approximately 60 ± 20 min. Figure 2 shows the number of hours dedicated to PT goals in the whole sample of PwND. The most selected PT goal for PwND is “walking” (34.9%), followed by “manipulation” (12.8%) and “balance” (11.1%). 

A subgroup analysis was carried out considering the number of hours per PT goals in each pathology (PwST, PwMS, and PwPD). Histograms with prevalence data are contained in the Supplementary Materials (Figures S2–S4).

A total of 3164 h of PT were dedicated to PwST (Figure S2). The most selected goal was “walking” with 1106 h (35%), followed by “manipulation” with 416 h (13.1%) and “balance” with 398 h (12.6%), while the least selected goals were “joint mobility”, “cardiorespiratory fitness”, “cognitive function”, and “QoL”.

In PwMS, the most selected goal was “walking” with 350 h (34.3%) out of 1019 (Figure S3), followed by “manipulation” with 182 h (17.9%), while the third was “muscle strength” with 86 h (8.4%). Conversely, goals such as “ADL”, “outdoor mobility”, or “pain” and “tertiary prevention” were rarely chosen.

Considering PwPD, the most selected goal was “walking” with 213 out of 610 h, representing 34.9% of the total treatment time, followed by “balance” with 70 h (11.5%) and “outdoor mobility” with 68 h (11.1%) (Figure S4). Again, “pain”, “cardiorespiratory fitness” and “QoL” were less considered.

### 3.4. Prevalence of PT Intervention

**Figure 3:**
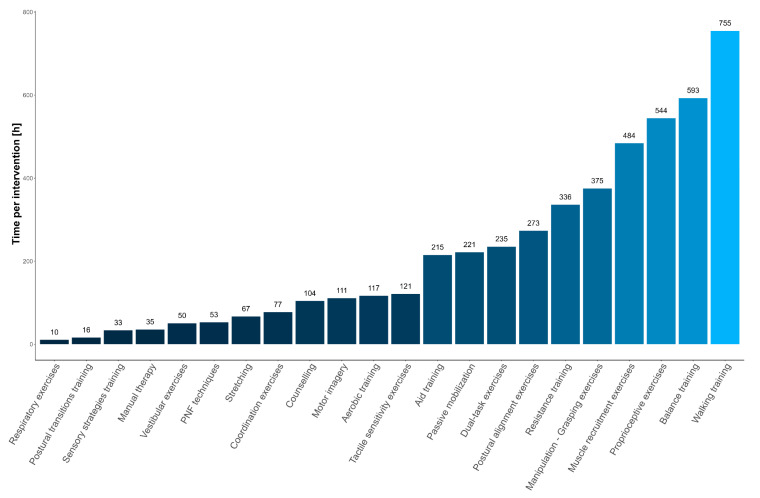
Number of hours per PT intervention in PwND.

The PT intervention most frequently proposed in the whole sample was “walking training” with 755 h (15.8%), followed by “balance training” with 593 h (12.4%). Figure 3 shows the number of hours dedicated to each PT intervention in PwND. 

A subgroup analysis was carried out considering the number of hours per PT intervention in each pathology (PwST, PwMS, and PwPD). Histograms with prevalence data are contained in the Supplementary Materials (Figures S5–S7).

Considering the most selected PT intervention for PwST, 439 h (13.9%) have been dedicated to “walking training”, followed by 425 h (13.4%) to “balance training” and 420 h (13.3%) to “proprioceptive exercises” (Figure S5). Conversely, “PNF techniques”, “sensory strategies training”, “manual therapy”, and “postural transitions training” were rarely used.

In PwMS, the most selected intervention was “walking training” with 171 h (16.8%), followed by “manipulation—grasping exercises” with 156 h (15.3%) (Figure S6). The least used interventions in this population were “postural alignment exercises”, “coordination exercises”, “stretching”, “dual-task exercises”, “aid training”, “manual therapy”, “PNF techniques”, “counselling” and “motor imagery”. 

Considering the PD population, “walking training” was the most selected PT intervention with 123 h (20.2%), followed by “balance training” with 80 h (13.1%) and “dual-task exercises” with 57 h (9.3%) (Figure S7). Conversely, interventions such as “counselling”, “aerobic training”, “passive mobilization”, “aid training”, “manipulation—grasping exercises”, “stretching”, and “vestibular exercises” were less considered.

### 3.5. PT Intervention Effectiveness

Interventions’ effectiveness (Table 4) was verified on the whole sample of PwND by the outcome measures previously described: MDGI, 2MWT, BBT, and MBI. 

The majority of the sample experienced a clinically significant improvement in all outcome measures assessing gait and balance function. In addition, more than one-third of the subjects obtained a clinically significant improvement in scales assessing manipulation function.

The following information in the text refers to subgroups, but the PT intervention effectiveness for each subgroup is also reported in Table S2 of the Supplementary Materials.

Most of the PwST were classified as improved on the clinical scales for gait and ADL, while less than half of the subjects improved on tests for upper-limb function. We identified a 2MWT mean improvement of 33.6 ± 34.9 m (N = 99 subjects; 68 improved) and an MDGI mean improvement of 16.1 ± 14.2 points (N = 98 subjects; 70 improved). In addition, BBT mean improvement was 6.3 ± 9.4 number of blocks (N = 88 subjects; 37 improved), and MBI mean improvement corresponded to 24 ± 22.5 points (N = 109 subjects; 72 improved). 

As far as PwMS are concerned, less than half of the sample was classified as improved on the clinical scales for gait and manipulation, while only 4 subjects were classified as having improved in ADLs. PwMS showed a 2MWT mean improvement of 2.4 ± 29.1 m (N = 39 subjects; 10 improved). Similarly, MDGI mean improvement was 2.7 ± 6.1 points (N = 34 subjects; 7 improved). BBT showed mean improvement of 2.9 ± 6.3 number of blocks (N = 40 subjects; 13 improved). MBI showed mean improvement of 4.1 ± 7.5 points (N = 43 subjects; 4 improved). 

Finally, in subjects with PD, about one-quarter of them improved on the scales for gait and upper limb, and only 3 subjects in the ADLs. 2MWT mean improvement in PwPD was 5 ± 19.2 m (N = 22 subjects; 5 improved), and MDGI mean improvement corresponded to 2.2 ± 5.3 points (N = 28 subjects; 6 improved). Considering the upper-limb test, BBT showed a mean improvement of 2.5 ± 7.6 number of blocks (N = 30 subjects; 6 improved), while MBI showed a mean improvement of 2.6 ± 14.2 points (N = 30 subjects; 3 improved).

## 4. Discussion

This paper aimed to investigate the content and effectiveness of PT for PwND through PBE methodology. This observational approach does not disrupt the treatment setting and assesses the full range of interventions used across all participants in a real-life context [23,25]. Data from six hospitals and three rehabilitation centers in Italy were collected and analyzed to describe the prevalence of PT goals and interventions in everyday clinical practice. Moreover, we investigated the global impact of the interventions on functional outcomes. 

To address the identified gap of knowledge, we attempted to classify the PT contents during everyday clinical practice in PwND, also providing quantitative data, in terms of hours of PT treatment. We found that the most used PT interventions were those aimed at improving gait function and balance performances. Moreover, we reported the effectiveness of PT on rehabilitation outcomes. 

### 4.1. Characteristics of the Clinical Units

A positive aspect of this study is that data were collected from a variety of settings, both hospitals and rehabilitation centers. This made it possible to increase the generalizability of the results, in accordance with the PBE methodology. However, the type of PT setting and clinical units’ characteristics were relevant in determining the sample of subjects who participated in the study. Indeed, patients with diseases characterized by an acute event have been mainly recruited in hospitals and treated as inpatients, while those with chronic-degenerative diseases tend to be treated in rehabilitation centers as outpatients, except for PwMS. Even if MS is a chronic degenerative disease, PwMS were mostly recruited in an inpatient setting.

### 4.2. Demographic and Clinical Characteristics of the Sample

This is the first study investigating the content of PT sessions in several neurological disorders. Other studies with similar purposes were focused only on one neurological disorder, such as spinal cord injuries [12,39], stroke [40,41], multiple sclerosis [13,15], and Parkinson’s disease [42]. The inclusion of multiple neurological disorders may have reduced the specificity of the results but allowed broader data collection and comparison between different conditions.

In our study, PwST accounted for most of the total sample, and most of them were treated in an inpatient setting as they were mainly in the early subacute phase of the disease [43]. ST is the leading cause of disability in Italy with a prevalence of 307/100,000 inhabitants [44,45]. On the other hand, PwMS are about half of the PwST recruited in this study. The proportion between PwST and PwMS in our sample is representative of the situation nationwide regarding the prevalence of these two diseases. In fact, the prevalence of PwMS in Italy is 179/100,000 inhabitants [46]. PwPD were the least represented population within our sample, and the prevalence of PwPD in this study is not representative of the situation nationwide compared to the other pathologies. According to epidemiological studies, PD is the most common neurodegenerative movement disorder in Italy with a prevalence rate of 197/100,000 inhabitants increasing in the elderly population [47].

From a clinical point of view, PwST and PwMS showed a moderate-to-severe level of disability in both walking and manipulation functional outcomes at baseline assessment. On the other hand, PwPD showed better baseline scores in clinical scales evaluating upper- and lower-limb function. Similarly, regarding functional independence during ADL, PwST showed a severe limitation, while PwPD showed a mild limitation.

### 4.3. Prevalence of PT Goals

The average duration of each PT program was more than 15 h of intervention, and this is in line with Rasova et al. [15], who reported that South and West European regions provide longer duration of therapy (median 15 h) than East and North regions (around 10 h).

In accordance with the ICF classification, the most selected goals were those related to activities and participation, in particular those related to the execution of a task or action by an individual (e.g., walking, manipulation, postural transitions) [48,49]. Among the different activities, walking remains the focus. In fact, regardless of the duration of pathology and level of disability, most PwND identify walking as the motor function in which they perceive the most difficulty and therefore the most disabling deficit [50]. Altogether, less than a third of the total time has been dedicated to goals related to body function and body structures (e.g., muscle strength, joint mobility, sensory functions) except for “balance”, which is the only goal related to an ICF body function placed third in order of prevalence. Exploring differences and similarities between pathologies, we observed that “walking” and “manipulation” activities are the most selected goals in PwST and PwMS. It has already been mentioned that “walking” is a priority among neurorehabilitation goals; moreover, PwST and PwMS showed relevant upper-limb deficit as spasticity, muscle weakness, sensory loss, and abnormal motor synergies, which contribute to making upper-limb activity a rehabilitation priority [51,52]. In addition, physical therapists selected more goals related to functional activities such as “postural transition” and “stair climbing” in PwST, while the prevalence of impairment-related goals become more relevant in PwMS (e.g., muscle strength, balance, and joint mobility). In contrast, the number of hours devoted to “manipulation” is significantly lower in PwPD, while most of the time during PT sessions was dedicated to mobility-related activities and participation (e.g., “walking” and “outdoor mobility”). Finally, compared to PwST and PwMS, more hours were dedicated to the impairment-related goals (e.g., “balance”, “trunk control”, and “joint mobility”) in PwPD with respect to the total amount of hours analyzed. These results are in line with rehabilitation guidelines [53] for PwPD in which most of the PT interventions are devoted to improving gait, mobility, transfer, and physical capacity compared to manipulation.

### 4.4. Prevalence of PT Intervention

The interventions performed were analyzed by their prevalence and grouped according to the classification proposed by Hart et al. [54]. According to this, the possible targets of the interventions can be structural tissue properties, organ functions, skilled performances, and cognitive–affective representations; moreover, the possible mechanism of action and the active ingredients promoted by the physical therapist have been suggested. 

In our study, the interventions used for the greatest number of hours were those targeting skilled performances or activities. This is in line with Bode et al. [40], who studied PT contents in PwST and found that physical therapists spent more rehabilitation time on function-focused activities.

According to Hart et al. [54,55], “Walking training” along with “Manipulation—grasping exercises”, “postural transitions training”, “coordination exercises”, “motor imagery”, “aid training”, and “dual-task exercises” are used to improve performance or skill, in terms of speed, efficiency, or quality. The mechanism of action of these interventions is based on motor learning principles [56] with a mixture of implicit and explicit learning processes, and the essential ingredient is the facilitation of the recipient’s performance by the physical therapist’s guidance [54]. Physical therapists actively provide instructions, coaching during the performance, cues, feedback, and strategies or methods to promote skills improvement and generalization. Other widely used types of interventions are those aiming to improve the functioning of a body system or organ such as “proprioceptive or tactile sensitivity exercises”, “muscle recruitment exercises”, “resistance training”, “vestibular exercises”, “respiratory exercises”, and “aerobic training”. Their mechanisms of action require the up- or down-regulation promoted by the central nervous system and by organ metabolism to stimulate change in the functional output of a particular body system or organ [55]. Physical therapists actively provide methods to improve the performance of a part of the recipient (e.g., setting intensity and number of repetitions during resistance training). On the other hand, less used interventions were those aimed at altering the structural properties of an organic tissue in terms of size, shape, or flexibility. Interventions with these characteristics are “passive mobilization”, “stretching”, and “manual therapy”. These interventions work by exploiting the macro- or microscopic tissue remodelling processes thanks to the application of energy directly on the tissue. Finally, the least applied types of interventions were those that aimed to modify self-cognitive–affective representations and self-behaviour. This is in line with Rasova et al. [15], who reported that interventions aimed at modifying self-cognitive–affective representations and self-behaviour providing advice and information were less used in southern European regions (e.g., Italy) compared to northern European regions. A typical representation of this kind of intervention is “counselling”. This intervention aims to modify self-behaviour facilitating the acquisition and retaining of information for the patient or caregiver and uses semantic memory and affective information processing as a mechanism of action.

Analyzing the interventions used in the three neurological disorders separately, it is evident that they reflect the therapeutic needs of each population. Indeed, “walking training” and “manipulation—grasping exercises” are among the most widely used for PwST and PwMS. Similar findings have been found by Martinkova et al. [13], who reported that balance training and postural awareness, training for transfers and ambulatory abilities, muscle stretching, strengthening, and aerobic training are well-known interventions and the most frequently used in PwMS. In contrast, “manipulation—grasping exercises” are used very little in PwPD, while “walking training” remains fundamental, and “postural alignment exercises” or “dual-task exercises” are mostly used as recommended in this population [57,58].

### 4.5. PT Intervention Effectiveness

Most of the participants showed improvement in clinical tests; however, we considered only the MCID value to determine the intervention effectiveness. According to this, the PT interventions had a positive effectiveness in PwND. The effectiveness of the intervention on PwST subjects was relevant, although it must be considered that the majority of PwST subjects were recruited in the early-subacute phase of the disease and therefore easily subject to the spontaneous recovery due to endogenous plasticity typical of the acute-subacute phase [43]. Looking at the effectiveness of the intervention on chronic conditions such as MS and PD, we observed that the number of subjects who obtained a clinically relevant improvement was drastically reduced. In chronic degenerative diseases, it seems that the intervention acted mainly as a performance maintenance effect so as not to deteriorate further in walking and manipulation function. However, as previously highlighted, we considered only the MCID value or 20% improvement over baseline to determine the PT effectiveness on each individual subject. This approach seems to reduce the overall treatment effectiveness because it is considered as improved participants who obtained a relevant clinical improvement in the performance, excluding all those who improved in clinical score but without reaching the MCID value. This may have reduced treatment effectiveness even if it is recommended to use the MCID as a reference since, over the past decades in modern medicine, there has been a shift from statistical significance to clinical relevance when it comes to interpreting results from clinical trials [59].

### 4.6. Limitations and Future Perspectives

This study has limitations that should be presented. First, there could be a recruitment bias since participants were selected according to the availability of centers. In particular, PwMS and PwPD were almost all recruited from the IRCCS Fondazione Don Gnocchi, Centro di Santa Maria Nascente, and this may have led to a center-related effect considering both the management of PT interventions and treatment outcomes. In addition, PwPD are poorly represented compared with the prevalence of the disease in the population, and this may have reduced the generalizability of the results. Another limitation is related to the “PT Interventions Form.” The semi-structured interview was not filled out after each PT session but only at the end of the rehabilitation program to reduce the burden of assessments increasing the rate of response. This may have reduced the reliability of the collected information; a more detailed description of PT interventions is still missing along with a strong theoretical framework. Moreover, the tool is still preliminary and not comprehensive since it does not include all PT goals and interventions in neurorehabilitation. Its future implementation in clinical practice and the possibility to add missing goals or intervention by physical therapists will allow to define any missing components through a bottom-up process. In addition, the tool highlighted problems such as the tautological definition between goals and interventions. For example, in our study, the intervention “walking training” is tautological and redundant with the goal “walking” because the contents of the intervention are defined by the name of the goal itself. To avoid this problem, it is necessary to use a theoretical construct as a top-down process to guide the definition of the content of the intervention. A possible theoretical construct has been proposed by Hart et al. [54,55], which defines the interventions according to their targets, or measurable aspects of functioning they are intended to change; ingredients, or measurable clinician decisions and behaviors responsible for effecting changes; and the hypothesized mechanisms of action by which ingredients are transformed into changes in the target. Finally, we investigated the dose of the intervention in terms of duration and frequency but we have not quantified the parameters of the intervention in terms of the number of repetitions of the exercise, amount of workload, and level of challenge of the task. Studying these parameters will allow us to better understand the impact of PT intervention on outcomes and to define the content of PT. Moreover, actually, we cannot recommend a minimum beneficial daily amount in clinical practice [60], but future analysis should determine the amount of PT needed to reach an MCID in PwND.

## 5. Conclusions

This longitudinal observational study allowed us to explore the contents of PT within major neurological populations. The most relevant goals, in accordance with the ICF classification, were those related to functional activities, among which walking was found to be the most important. Conversely, participation-related goals were less considered. The PT interventions most used by physical therapists were those aimed at improving a specific skill or performance, followed by those aimed at improving organ or body system function, while interventions for the cognitive–affective sphere targeting educational aspects have hardly been considered. The results of this study confirm the need to develop an ad hoc taxonomy to classify PT interventions in neurorehabilitation.

## Figures and Tables

**Table 1:** Characteristics of the clinical units (No. of participants).

Clinical Units	Type	PwND	ST	MS	PD	Setting
FDG (Milan)	Rehabilitation center	82	19	40	23	In = 59 (72%)
SPC (Milan)	Hospital	35	35	-	-	In = 32 (91.4%)
AIAS (Milan)	Rehabilitation center	13	5	2	6	Out = 13 (100%)
ORT (Ancona)	Hospital	17	17	-	-	In = 15 (88.2%) Out = 2
OPS (Genoa)	Hospital	9	6	-	3	In = 6 (66.6%)
IDO (Pescara)	Rehabilitation center	11	4	4	3	Out = 11 (100%)
ASUGI (Trieste)	Hospital	6	6	-	-	In = 6 (100%)
PSC (Turin)	Hospital	14	14	-	-	In = 14 (100%)
OSC (Venice)	Hospital	15	13	2	-	In = 15 (100%)

Abbreviation: FDG, IRCCS Fondazione Don Gnocchi, Centro di Santa Maria Nascente; SPC, ASST Santi Paolo e Carlo; AIAS; Associazione Italiana Assistenza Spastici; ORT, AOU Ospedali Riuniti Torrette; OPS, IRCCS Ospedale Policlinico San Martino; IDO, Istituto Don Orione; ASUGI, Azienda Sanitaria Universitaria Giuliano Isontina; PSC, Presidio Sanitario San Camillo; OSC, IRCCS Ospedale San Camillo; PwND, People with Neurological Disorders; MS, Multiple Sclerosis; PD, Parkinson’s Disease; ST, Stroke; In, Inpatient; Out, Outpatient.

**Table 2:** Demographic characteristics of the sample (No. of participants and %; mean and SD).

Subjects	Age (Years)	Gender	Setting
PwND N = 202 (100%)	65.9 (13)	F = 96 (47.5%)	In = 147 (72.8%)
ST N = 119 (58.9%)	67.4 (12.8)	F = 49(41.2%)	In = 101 (84.9%)
MS N = 48 (23.8%)	57.1 (12)	F = 29 (60.4%)	In = 34 (70.8%)
PD N = 35 (17.3%)	73.1 (8)	F = 18 (51.4%)	In = 12 (34.3%)

Abbreviations: PwND, People with neurological disorders; ST, Stroke; MS, Multiple Sclerosis; PD, Parkinson’s disease; In, Inpatient; Out, Outpatient; F, Female; M, Male; N, Number of subjects.

**Table 3:** Clinical characteristics at baseline assessment (No. of participants; mean and SD).

Subjects	MDGI (Score)	2MWT (m)	BBT (n°)	MBI (Score)
PwND N = 202	N = 178 21.7 (19)	N = 176 53.7 (51.7)	N = 176 26 (16.2)	N = 201 65.3 (26.4)
PwST N = 119	N = 107 16.8 (16.9)	N = 105 41.5 (43.7)	N = 94 20.2 (16.7)	N = 118 57.4 (23.9)
PwMS N = 48	N = 39 22.9 (19)	N = 44 59.37 (55.7)	N = 47 29.28 (12.7)	N = 48 73.79 (23.8)
PwPD N = 35	N = 32 36.6 (18.1)	N = 27 92.2 (54.8)	N = 35 37.4 (11.4)	N = 35 80.5 (28.2)

Abbreviations: PwND, People with neurological disorders; PwST, People with Stroke; PwMS, People with Multiple Sclerosis; PwPD, People with Parkinson’s disease; MDGI, Modified Dynamic Gait Index; 2MWT, 2-Minute Walking Test; BBT, Box and Block Test (most affected side); MBI, Modified Barthel Index; N, Number of subjects.

**Table 4:** PT intervention effectiveness on PwND.

Outcome Measure	T0 Mean (SD)	T1 Mean (SD)	Improvement Mean (SD)	Improved > MCID N (%)
2MWT (meters)	N = 176 53.7 (51.7)	N = 166 75 (49.5)	N = 160 22.1 (34.9)	N = 160 Improved = 83 (51.8%)
MDGI (score)	N = 178 21.7 (19)	N = 164 31.9 (21.8)	N = 160 10.8 (13.4)	N = 160 Improved = 83 (51.8%)
BBT (n°)	N = 176 26 (16.2)	N = 159 31.1 (16.6)	N = 158 4.70 (8.5)	N = 158 Improved = 56 (35.4%)
MBI (score)	N = 201 65.3 (26.4)	N = 182 80.7 (20.3)	N = 182 15.8 (21.2)	N = 182 Improved = 103 (56.6%)

Abbreviations: N, Number of subjects; SD, Standard deviation; MCID, Minimally clinically important difference; 2MWT, 2-Minute Walking Test; MDGI, Modified Dynamic Gait Index; BBT, Box and Block Test (most affected side); MBI, Modified Barthel Index.

## Data Availability

The datasets used and analyzed during the current study are available from the corresponding author upon reasonable request.

## Supplementary Materials

The following supporting information can be downloaded at: https://www.mdpi.com/article/10.3390/jcm12206483/s1, Figure S1: PT Interventions form; Figure S2: PT goals PwST; Figure S3: PT goals PwMS; Figure S4: PT goals PwPD; Figure S5: PT interventions PwST; Figure S6: PT interventions PwMS; Figure S7: PT interventions PwPD; Table S1: PT goals according to ICF; Table S2: PT intervention effectiveness in subgroups.
