# Supplementary material for: Classification and Quantification of Physical Therapy Interventions across Multiple Neurological Disorders: An Italian Multicenter Network
Source: J Clin Med. 2023 Oct 12;12(20):6483. doi: 10.3390/jcm12206483 (PMC10607918; doi:10.3390/jcm12206483)
Supplement: Supplementary file 1 [file jcm-12-06483-s001.zip › Figure S1_PT Interventions form.pdf]

Therapeutic goals	Priority (1,2,3)	Time %
**Functional - Activities**		sum of the 3 percentages MUST BE = 100%
Manipulation		
Walking		
Postural transitions		
Stair climbing		
ADL		
**Impairment**		
Pain		
Joint mobility		
Muscle strength		
Sensory functions		
Balance		
Trunk control		
Standing position		
Tertiary prevention		
Cardiorespiratory fitness		
Cognitive function		
Genitourinary functions		
**Participation**		
Outdoor mobility		
Quality of life		
**Other**		

LEGEND

Preliminary remark: Not all the possible therapeutic goals or treatments are already indicated in this form, please use the proper empty spaces to indicate what is not already included.

Priority: please indicate 3 main rehabilitative aims and give them a level of priority: **1(high), 2(medium), 3 (low).**

Time %: indicate the percentage of **time dedicated** to each selected goal, compared to total time of treatment.

IN/OUT PATIENT: indicate whether the setting of rehabilitative intervention is hospital-based **(IN)** or not **(OUT)**.

SUBJECT DATA	
**Name** ____________________________	
**Sessions frequence:** n° ______ times/week	
**Session lasting:**___ min	**IN/OUT PATIENT:** ☐ IN ☐ OUT
**Other Therapies:**	☐Speech/swallow rehabilitation ☐Occupational Therapy<br>☐Neuropsychologist ☐Massage Therapy<br>☐____________________ (Other)

Interventions	Goal 1	Goal 2	Goal 3
	% time	% time	% time
Aid training			
Aerobic training			
Postural alignment exercises			
Constraint induced movement therapy			
Counselling			
Tactile sensitivity exercises			
Dual-task execises			
Manipulation-grasping exercises			
Proprioceptive exercises			
Vestibular exercises			
Motor imagery			
Limphatic drainage			
Passive mobilization			
Muscle recruitment exercises			
Resistance training			
Stretching			
Manual therapy			
Instrumental physical therapies (specify________________________)			
Walking training			
Balance training			
Sensory strategies training			
Myofascial release			

Note: Indicate upt to 5 interventions type for each goal. Specify the percentage time dedicated to each intervention. The sum must be 100% for each goal.
