# Supplementary material for: Classification and Quantification of Physical Therapy Interventions across Multiple Neurological Disorders: An Italian Multicenter Network
Source: J Clin Med. 2023 Oct 12;12(20):6483. doi: 10.3390/jcm12206483 (PMC10607918; doi:10.3390/jcm12206483)
Supplement: Supplementary file 1 [file jcm-12-06483-s001.zip › Figures S2-S7.pdf]

Journal of
Clinical Medicine

MDPI

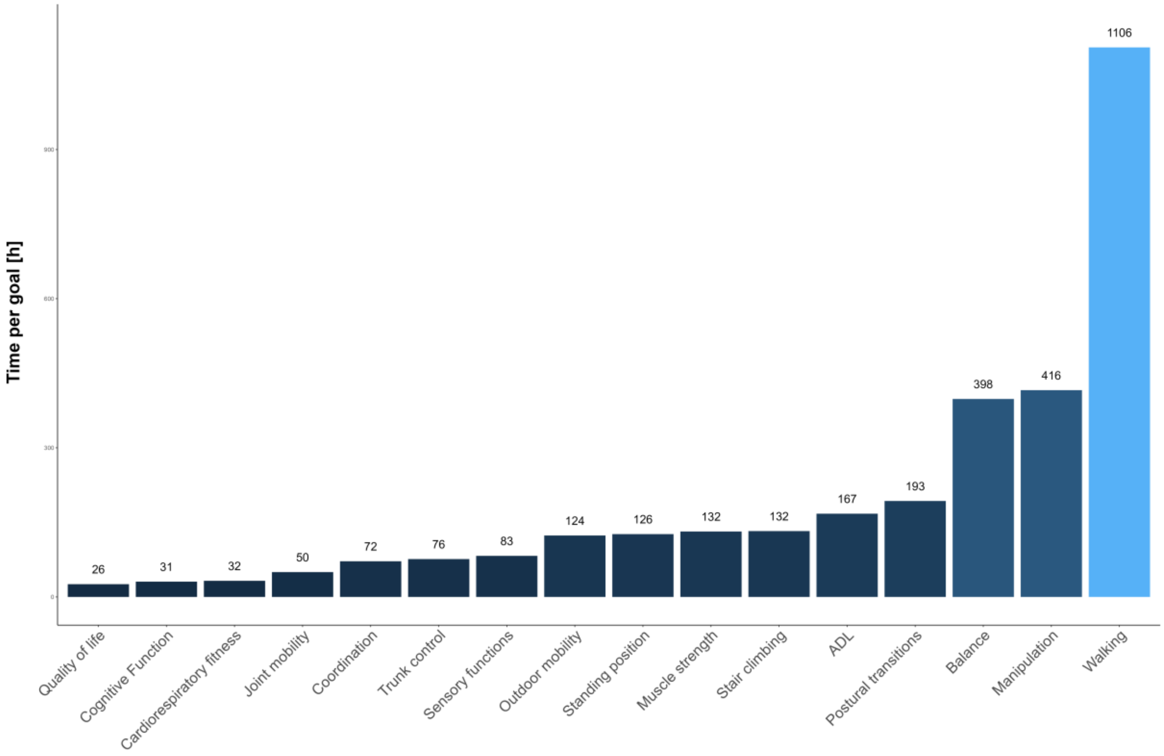

Figure S2. PT goals PwST.

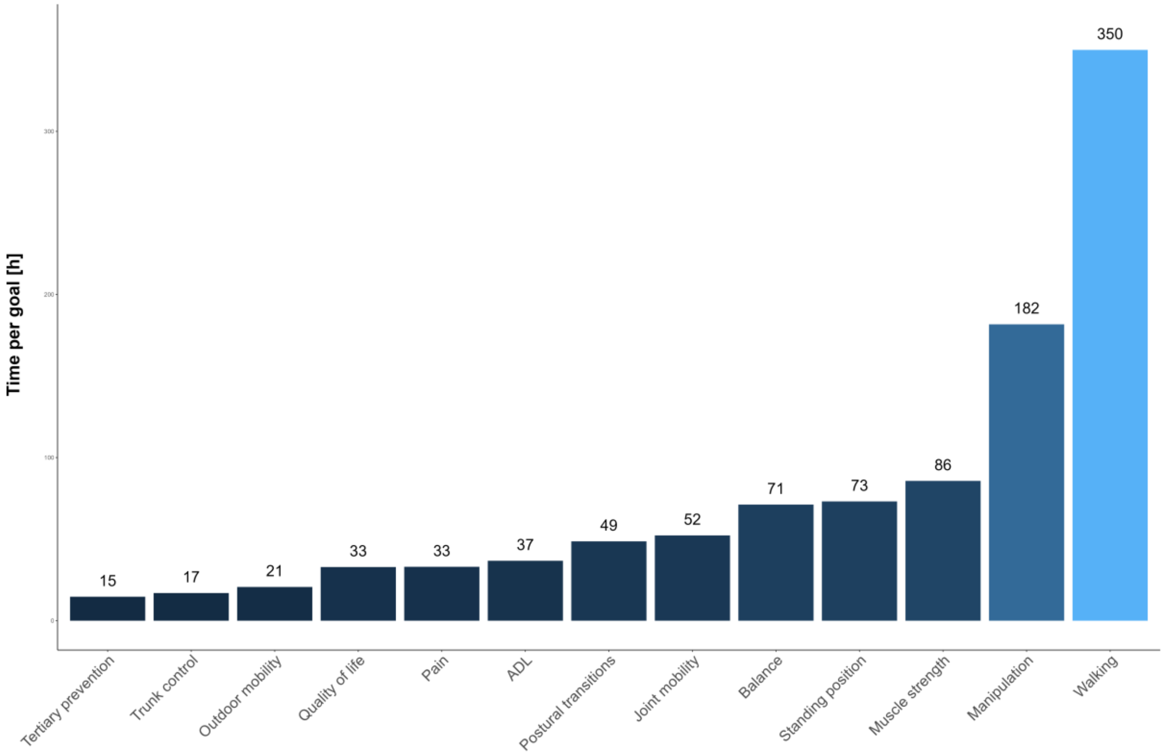

Figure S3. PT goals PwMS.

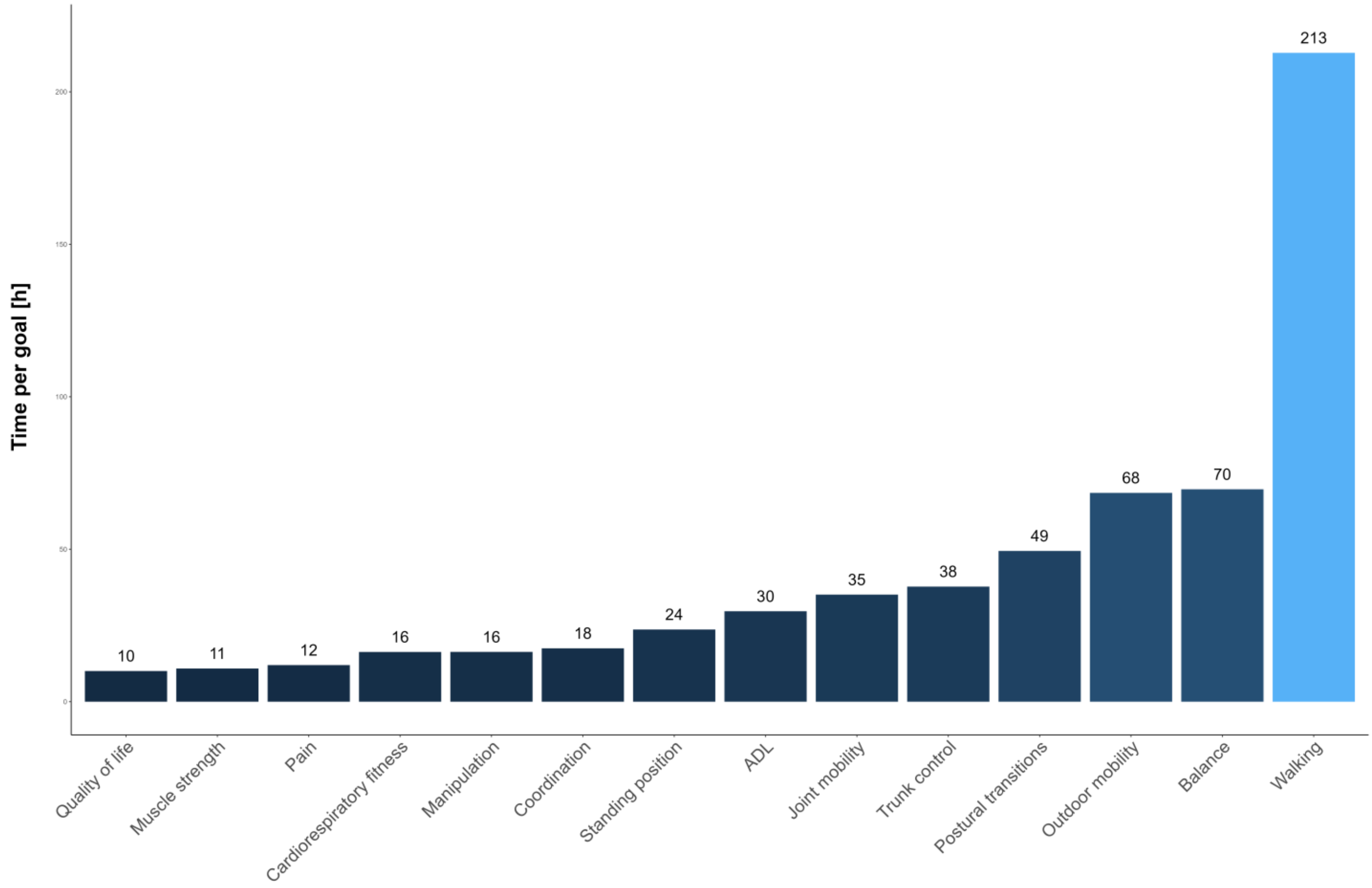

Figure S4. PT goals PwPD.

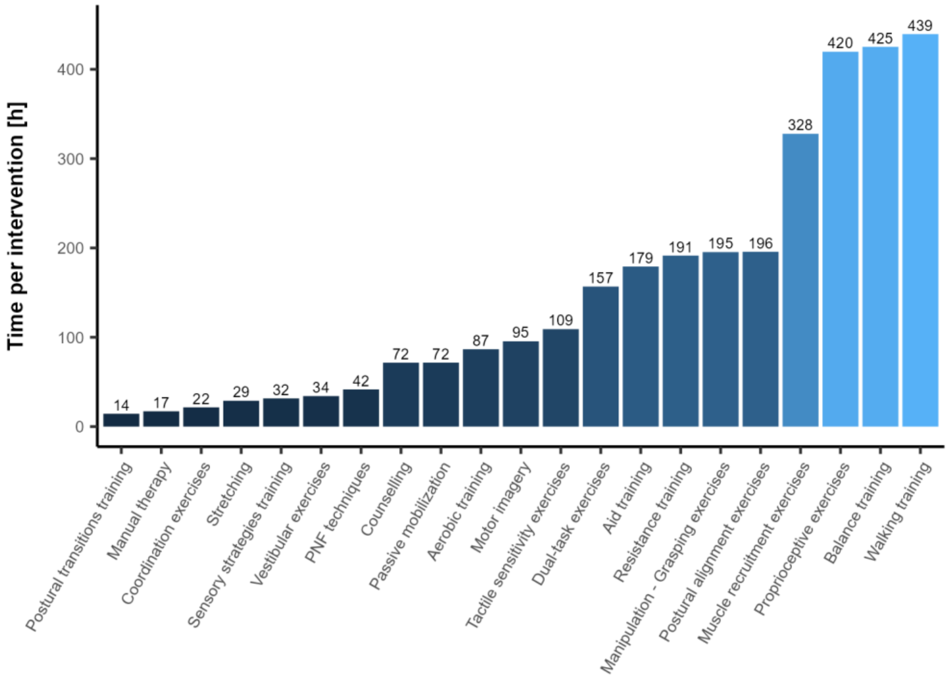

Figure S5. PT interventions PwST.

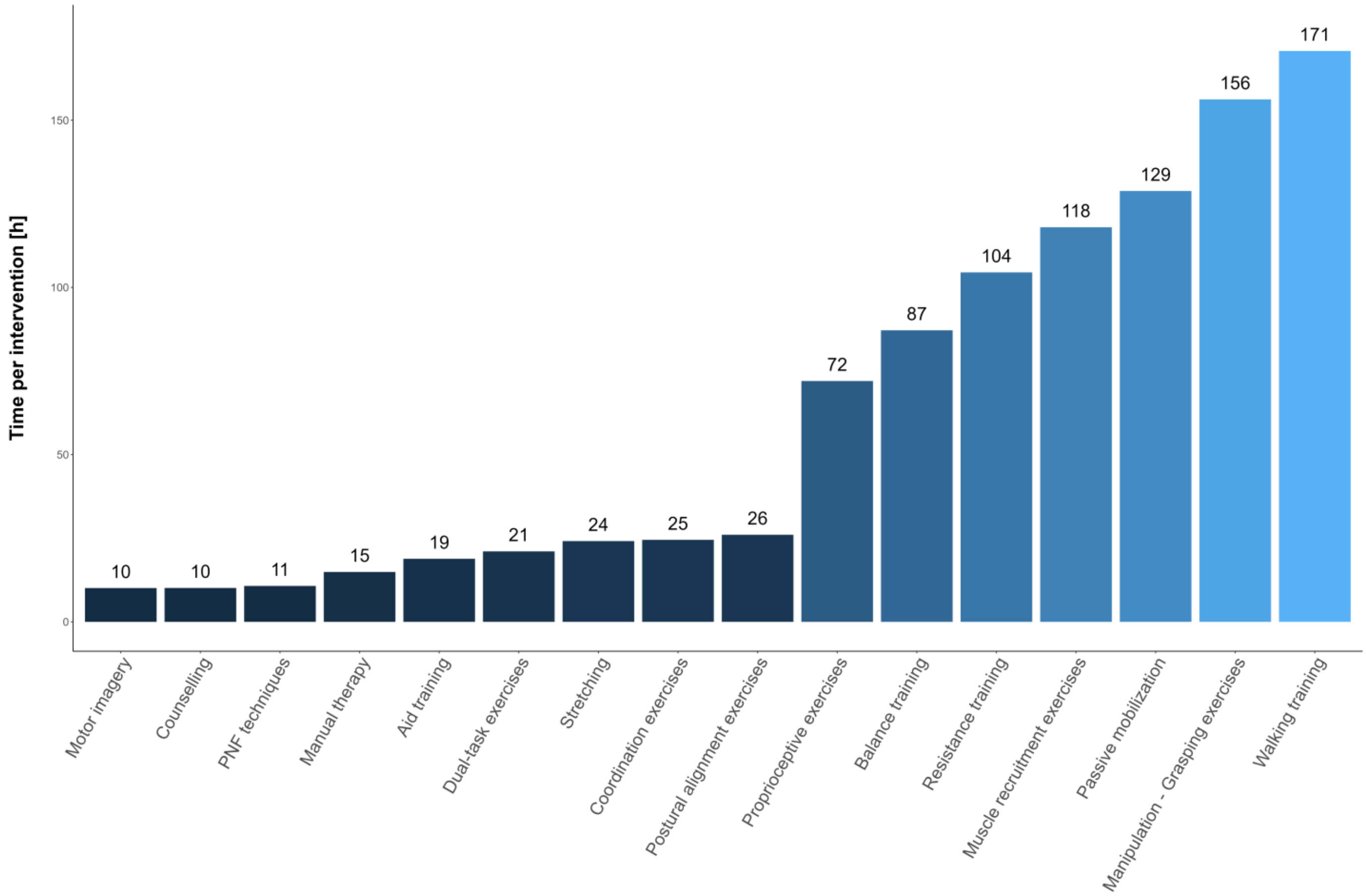

Figure S6. PT interventions PwMS.

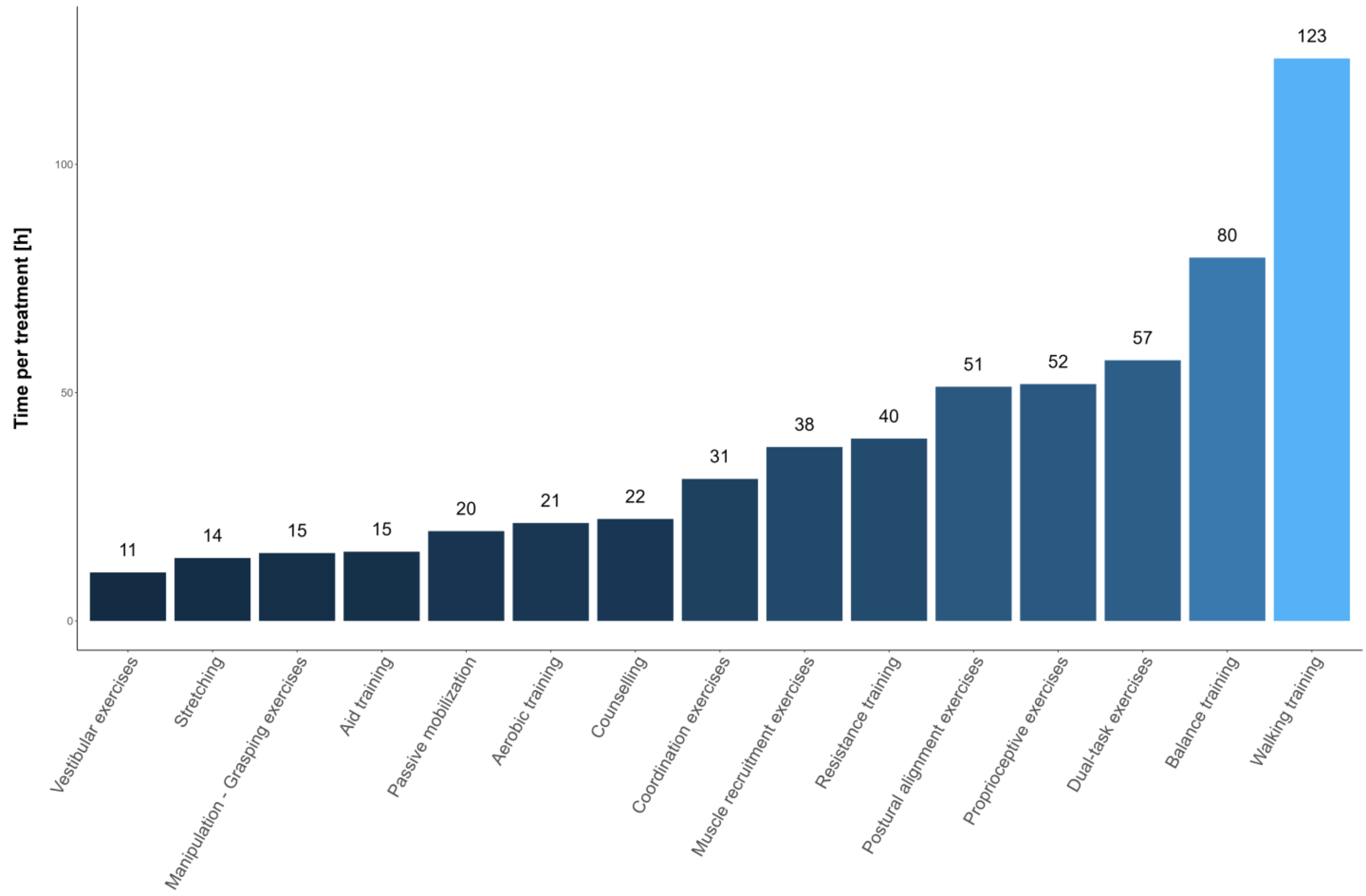

Figure S7. PT interventions PwPD.
