# Supplementary material for: Classification and Quantification of Physical Therapy Interventions across Multiple Neurological Disorders: An Italian Multicenter Network
Source: J Clin Med. 2023 Oct 12;12(20):6483. doi: 10.3390/jcm12206483 (PMC10607918; doi:10.3390/jcm12206483)
Supplement: Supplementary file 1 [file jcm-12-06483-s001.zip › Table S1_PT Goals according to ICF.pdf]

Table S1. List of Physical Therapy Goals matched with ICF categories from generic, rehabilitation, and neurological core set.

THERAPEUTIC GOALS	ICF CATEGORIES
**ACTIVITIES**	
Manipulation	Fine hand use (d440); Hand and arm use (d445); Lifting and carrying objects (d430)
Walking	Walking (d450)
Postural transitions	Changing basic body position (d410); Repositioning oneself (d420)
Standing position	Standing (d4104); Maintaining a standing position (d4154)
Stair climbing	Moving around within the home (d4600); Moving around within buildings other than the home (d4601);
ADL	Undertaking a single task (d210); Undertaking multiple tasks (d220); Carrying out daily routine (d230); Washing oneself (d510); Doing housework (d640); Caring body parts (d520); Toileting (d530); Dressing (d540); Eating d (d550); Drinking (d560); Preparing Meals (d630)
**PARTICIPATION**	
Outdoor mobility	Moving around outside the home and other buildings (d4602); Using transportation (d470)
Quality of life	Acquiring skills (d155); Solving problems (d175); Making decisions (d177); Handling stress and other psychological demands (d240); Basic interpersonal relationships (d710); Complex interpersonal interactions (b720); Informal social relationships (d750); Human Rights (d940); Remunerative employment (d850); Non-remunerative employment (d855); Community life (d910); Recreation and leisure (d920).
**IMPAIRMENT**	
Pain	Sensation of pain (b280)
Joint mobility	Mobility of joint functions (b710); Stability of joint functions (b715); Mobility of bone functions (b720);
Muscle strength	Muscle power functions (b730); Muscle tone functions (b735); Muscle endurance functions (b740); Motor reflex function (b750); Control of voluntary movement functions (b760)
Sensory functions	Proprioceptive function (b260); Sensations related to muscles and movement functions (b780); Touch function (b265); Sensory functions related to temperature and other stimuli (b270); Sensations related to the skin (b840)
Balance	Vestibular function (b235); Proprioceptive function (b260); Seeing function (b210); Involuntary movement reaction functions (b755)
Trunk Control	Power of isolated muscle and muscle groups (b7300); Power of muscles of the trunk (b7305); Tone of muscles of trunk (b7355).
Tertiary prevention	Mobility of joint functions (b710); Mobility of bone functions (b720); Muscle tone functions (b735); Protective function of the skin (b810); Repair function of the skin (b820).
Cardiorespiratory fitness	Heart functions (b410); Blood pressure functions (b420); Respiration functions (b440); Respiratory muscle functions (b445); Additional respiratory functions(b450); Exercise tolerance functions (b455).
Cognitive function	Orientation functions (b114); Attention functions (b140); Memory Functions (b144); Psychomotor functions (b147); Perceptual functions (b156); Thought functions (b160); Higher-level cognitive functions (b164); Mental function of sequencing complex movements (b176); Experience of self and time functions (b180).
Genitourinary functions	Defecation functions (b525); Urination functions (b620); Sexual Functions (b640).

Abbreviations: ICF, International Classification of Functioning , disability and health; ADL, Activities of daily living
