# Supplementary material for: Classification and Quantification of Physical Therapy Interventions across Multiple Neurological Disorders: An Italian Multicenter Network
Source: J Clin Med. 2023 Oct 12;12(20):6483. doi: 10.3390/jcm12206483 (PMC10607918; doi:10.3390/jcm12206483)
Supplement: Supplementary file 1 [file jcm-12-06483-s001.zip › Table S2_PT intervention effectiveness in subgroups.pdf]

Table S2. PT intervention effectiveness in subgroups.

Pathology	Outcome	T0 Mean (SD)	T1 Mean (SD)	Improvement Mean (SD)	MCID
PwST	2MWT	N= 105<br>41.5 (43.7)	N= 103<br>73.4 (46.4)	N= 99<br>33.6 (34.9)	Improved = 68<br>Not improved = 31
	MDGI	N= 107<br>16.8 (16.9)	N= 101<br>32 (19.9)	N= 98<br>16.1 (14.2)	Improved = 70<br>Not improved = 28
	BBT	N= 94<br>20.2 (16.7)	N= 89<br>26.7 (18.1)	N= 88<br>6.3 (9.4)	Improved = 37<br>Not improved = 51
	MBI	N= 118<br>57.4 (23.9)	N= 109<br>80.6 (19.1)	N= 109<br>24 (22.5)	Improved = 72<br>Not improved = 37
PwMS	2MWT	N= 44<br>59.4 (55.7)	N= 40<br>65.5 (50.7)	N= 39<br>2.4 (29.1)	Improved = 10<br>Not improved = 29
	MDGI	N= 39<br>22.9 (19)	N= 35<br>25.6 (21.5)	N= 34<br>2.7 (6.1)	Improved = 7<br>Not improved = 27
	BBT	N= 47<br>29.3 (12.7)	N= 40<br>33.5 (13.1)	N= 40<br>2.9 (6.3)	Improved = 13<br>Not improved = 27
	MBI	N= 48<br>73.8 (23.8)	N= 43<br>78.5 (22.6)	N= 43<br>4.1 (7.5)	Improved = 4<br>Not improved = 39
PwPD	2MWT	N= 27<br>92.2 (54.8)	N= 23<br>98.7 (55.3)	N= 22<br>5 (19.2)	Improved = 5<br>Not improved = 17
	MDGI	N= 32<br>36.6 (18.1)	N= 28<br>39.6 (18)	N= 28<br>2.2 (5.31)	Improved = 6<br>Not improved = 22
	BBT	N= 35<br>37.4 (11.4)	N= 30<br>40.93 (10.7)	N= 30<br>2.5 (7.6)	Improved = 6<br>Not improved = 24
	MBI	N= 35<br>80.5 (28.2)	N= 30<br>84.00 (21.6)	N= 30<br>2.6 (14.2)	Improved = 3<br>Not improved = 27

Abbreviations: PwST, People with Stroke; PwMS, People with Multiple Sclerosis; PwPD, People with Parkinson's Disease; N, Number of subjects; SD, Standard deviation; MCID, Minimally clinically important difference; 2MWT, 2 Minute Walking Test; MDGI, Modified Dynamic Gait Index; BBT, Box and Block Test (most affected side); MBI, Modified Barthel Index.
